# Engineering a “muco‐trapping” ACE2‐immunoglobulin hybrid with picomolar affinity as an inhaled, pan‐variant immunotherapy for COVID‐19

**DOI:** 10.1002/btm2.10650

**Published:** 2024-02-07

**Authors:** Karthik Tiruthani, Carlos Cruz‐Teran, Jasper F. W. Chan, Alice Ma, Morgan McSweeney, Whitney Wolf, Shoufeng Yuan, Vincent K. M. Poon, Chris C. S. Chan, Lakshmi Botta, Brian Farrer, Ian Stewart, Alison Schaefer, Jasmine Edelstein, Priya Kumar, Harendra Arora, Jeff T. Hutchins, Anthony J. Hickey, Kwok‐Yung Yuen, Samuel K. Lai

**Affiliations:** ^1^ Division of Pharmacoengineering and Molecular Pharmaceutics University of North Carolina at Chapel Hill Chapel Hill North Carolina USA; ^2^ State Key Laboratory of Emerging Infectious Diseases, Carol Yu Centre for Infection, Department of Microbiology, Li Ka Shing Faculty of Medicine The University of Hong Kong Pokfulam, Hong Kong Special Administrative Region China; ^3^ Centre for Virology, Vaccinology and Therapeutics Hong Kong Science and Technology Park Hong Kong Special Administrative Region China; ^4^ UNC/NCSU Joint Department of Biomedical Engineering University of North Carolina at Chapel Hill Chapel Hill North Carolina USA; ^5^ Inhalon Biopharma, Inc. Morrisville North Carolina USA; ^6^ RTI International Research Triangle Park North Carolina USA; ^7^ Department of Anesthesiology, School of Medicine University of North Carolina Chapel Hill North Carolina USA; ^8^ Department of Microbiology and Immunology University of North Carolina at Chapel Hill Chapel Hill North Carolina USA

**Keywords:** ACE2, antibody, COVID‐19, drug delivery, immunodecoy, inhaled delivery, monoclonal antibody, SARS‐CoV‐2

## Abstract

Soluble angiotensin‐converting enzyme 2 (ACE2) can act as a decoy molecule that neutralizes severe acute respiratory syndrome coronavirus 2 (SARS‐CoV‐2) by blocking spike (S) proteins on virions from binding ACE2 on host cells. Based on structural insights of ACE2 and S proteins, we designed a “muco‐trapping” ACE2‐Fc conjugate, termed ACE2‐(G_4_S)_6_‐Fc, comprised of the extracellular segment of ACE2 (lacking the C‐terminal collectrin domain) that is linked to mucin‐binding IgG1‐Fc via an extended glycine‐serine flexible linker. ACE2‐(G_4_S)_6_‐Fc exhibits substantially greater binding affinity and neutralization potency than conventional full length ACE2‐Fc decoys or similar truncated ACE2‐Fc decoys without flexible linkers, possessing picomolar binding affinity and strong neutralization potency against pseudovirus and live virus. ACE2‐(G_4_S)_6_‐Fc effectively trapped fluorescent SARS‐CoV‐2 virus like particles in fresh human airway mucus and was stably nebulized using a commercial vibrating mesh nebulizer. Intranasal dosing of ACE2‐(G_4_S)_6_‐Fc in hamsters as late as 2 days postinfection provided a 10‐fold reduction in viral load in the nasal turbinate tissues by Day 4. These results strongly support further development of ACE2‐(G_4_S)_6_‐Fc as an inhaled immunotherapy for COVID‐19, as well as other emerging viruses that bind ACE2 for cellular entry.

**Translational Impact Statement**

To overcome the immune‐evasive nature of severe acute respiratory syndrome coronavirus 2 (SARS‐CoV‐2) that has now escaped all monoclonal antibodies initially advanced into the clinic and that received emergency use authorization, we have developed here a “muco‐trapping” ACE2‐Fc decoy. This molecule not only can bind and neutralize SARS‐CoV‐2 with picomolar affinities, but more importantly is not at risk of viral escape. To address the burdens of intravenous infusion on the healthcare system and inconvenience to patients, we have further advanced a formulation that can be stably nebulized with a hand‐held device. The end result is a treatment that can be dosed in the convenience of one's own home and is expected to block the spread of SARS‐CoV‐2 and other ACE2‐targeted respiratory infections locally, as well as quickly clear trapped virions from the respiratory tract.

## 1 INTRODUCTION

Despite the ready availability of numerous vaccines against COVID‐19, there continues to be major demand for safe and effective antivirals that are easily administered, particularly among high‐risk individuals, and do not suffer risk for drug–drug interactions.^1^ Furthermore, in light of the ever‐shifting landscape of immune evasive variants of severe acute respiratory syndrome coronavirus 2 (SARS‐CoV‐2), there is a clear need to develop pan‐variant antiviral therapies that may be broadly effective against a spectrum of coronaviruses or other respiratory viruses.

**FIGURE 1:**
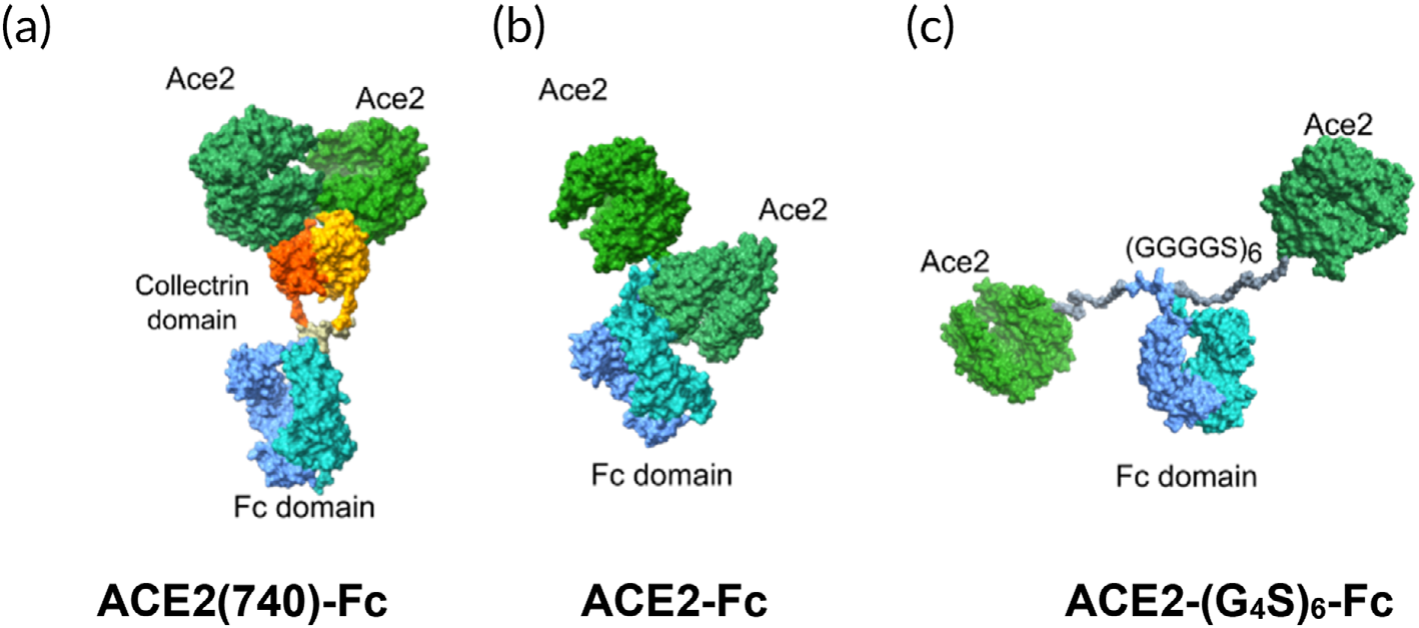
Computational prediction of hypothetical structures of different ACE2 decoys. (a) ACE2‐Fc fusion comprised of the entire ACE2 molecule including the collectrin domain linked to IgG1‐Fc (ACE2(740)‐Fc).^1^ (b) ACE2‐Fc fusion containing the extracellular domain of ACE2 without collectrin domain, linked to Fc with no flexible linker. (c) ACE2 fragment without collectrin domain linked to human IgG1‐Fc via a 30 amino acid glycine‐serine flexible linker (ACE2‐(G_4_S)_6_‐Fc). The flexible linker, shown in gray, allows for much longer reach of the two ACE2 domains. UCSF Chimera 1.14^2^ was used to generate all the protein models, and UCSF Chimera X 1.1^3^ was used to render the model for publication. ACE2‐Fc and ACE2‐(G_4_S)_6_‐Fc were constructed using models 6M17 for ACE2,^4^ 1HZH for human IgG,^5^ and 1EIB for GGGGS linker.^6^ ACE2‐Fc and ACE2‐(G_4_S)_6_‐Fc bound to S protein were generated by matching the ACE2 of ACE2‐Fc and ACE2‐(G_4_S)_6_‐Fc with the RBD‐bound ACE2 in the “all‐up” S protein model 7A98.^7^ The predicted 3D model of ACE2‐Fc with collectrin domain was modified from Reference 1. ACE2, angiotensin‐converting enzyme 2; RBD, receptor binding domain.

SARS‐CoV‐2 gains entry into cells when the receptor binding domain (RBD) of its spike protein (S) binds to angiotensin‐converting enzyme 2 (ACE2) on the target cell's surface,^2^ similar to SARS‐CoV‐1^3^ and NL63‐CoV.^4^ A number of research groups have taken advantage of SARS‐CoV‐2's ACE2‐tropism to develop ACE2‐Fc decoys that can neutralize the virus,^5^, ^6^, ^7^ typically by linking either the entire ACE2 molecule (residues 18–740, which includes the self‐dimerizing collectrin domain) to human IgG1‐Fc,^8^, ^9^, ^10^, ^11^ or linking simply the extracellular segment of ACE2 without the C‐terminal collectrin domain (residues 18–614) to human IgG1‐Fc (Figure 1a,b).^5^, ^12^, ^13^, ^14^, ^15^ Unfortunately, since S‐proteins only bind ACE2 with modest affinity, the neutralizing potencies of such ACE2 decoys are also rather modest.^5^, ^6^ To overcome the limited affinity, several groups have engineered higher affinity ACE2 variants by random mutagenesis and selection using yeast surface display.^5^, ^6^, ^8^ Nevertheless, the directed evolution strategy leaves open the possibility of an escape SARS‐CoV‐2 variant that binds wildtype ACE2 receptor but not the mutated ACE2 molecule. A mutated ACE2 may also have lower affinity to other viruses that bind ACE2. Alternatively, it is possible to increase the overall avidity of the molecule to the virus by increasing the valency of ACE2 binding domains per molecule^12^, ^16^, ^17^, ^18^; however, this would likely result in much larger constructs that are in turn more difficult to manufacturer, stabilize, and formulate. We thus sought a strategy to improve the binding of wildtype ACE2 to the virus without changing the sequence of ACE2 from the native human protein, while maintaining the same binding domain valency as conventional IgGs.

Cryo‐electron microscopy of SARS‐CoV‐1 showed that ~50–100 S proteins are present on the virus surface, with an average spacing of ~15 nm.^18^ The trimeric form of the S‐protein spike also results in a large distance between any two of the three S protein monomers within an individual spike trimer. Such distances limit the ability for a single antibody or ACE2‐conjugate to simultaneously bind to two distinct S‐proteins. These structural insights motivated us to improve the binding of our ACE2‐decoy by tuning the presentation of the two ACE2 domains. Specifically, we replaced the VH‐CH1 domain of the standard IgG1 Fab with the extracellular domain of ACE2 only (residues 18–614; no self‐dimerizing collectrin domain), and further introduced an extended 30‐amino acid flexible linker between the ACE2 fragment and CH2 in order to increase the reach of each of the two ACE2 arms, which should increase the probability of bivalent binding across spike trimers (Figure 1c).

SARS‐CoV‐2,^19^ just like SARS‐CoV‐1^20^ and NL63 and HKU1 coronaviruses,^4^, ^21^, ^22^, ^23^ infects strictly via the apical side of airway epithelium (i.e., airway lumen), and predominantly sheds progeny virions back into airway mucus (AM) overlaying the epithelium. This pathophysiology suggests that crosslinking virions to mucins in the AM may block the local spread of viral infections in the respiratory tract and facilitate rapid elimination of viruses by natural mucociliary or cough‐driven clearance.^24^, ^25^, ^26^ Building off of our previous discovery that the Fc domain of IgG1 possess a weak affinity to mucins that can enable potent trapping of viruses in mucus,^24^, ^27^, ^28^ we engineered here a “muco‐trapping” ACE2‐immunoglobulin construct (ACE2‐(G_4_S)_6_‐Fc) that binds different variants of SARS‐CoV‐2 S proteins, potently neutralizes different SARS‐CoV‐2 pseudovirus and live virus, effectively traps SARS‐CoV‐2 virus like particles in human AM, can be stably nebulized, and effectively reduces SARS‐CoV‐2 infections in hamsters. This molecule is now being actively advanced into clinical development as an inhaled treatment for ACE2‐binding pathogens, including SARS‐CoV‐2.

## 2 RESULTS

### 2.1 Designing ACE2‐(G_4_S) _6_‐Fc

To establish how ACE2 may associate with a trimeric Spike protein, we first generated the reported spike protein structures in the “three up” RBDs conformation.^29^ We then determined whether an ACE2‐Fc molecule could bind to two RBDs on the same S protein trimer (Figure S1a). When one of the two ACE2 domains on ACE2‐Fc engages any one of the three RBD in the spike trimer, the remaining ACE2 domain becomes oriented upwards, away from the S protein, due to the lack of flexibility and length in the hinge of IgG1 connecting ACE2 to Fc. Thus, it is not likely that the ACE2‐Fc can bind bivalently to two RBDs on the same Spike protein (or to two RBDs on two different Spike proteins on the virus). The same limitation holds for the conventional ACE2‐Fc encompassing the collectrin domain, since the dimerization of the collectrin domain limits the reach of the adjacent ACE2 fragments.

The estimated distances between RBDs within a spike trimer ranged from 60 to 100 Å when the three RBDs were in the “three‐up” conformation. To bridge the distance and add flexibility to the molecule, we introduced a (GGGGS)_6_ flexible linker with a length of ~10 nm between the extracellular ACE2 fragment and IgG1‐Fc to form bivalent ACE2‐(G_4_S)_6_‐Fc (Figure 1c, Figure S1b. Since the flexible linker is present on each of the two heavy chains, the two ACE2 fragments on ACE2‐(G_4_S)_6_‐Fc can theoretically span distances nearly twice that length, that is, up to ~20 nm.

### 2.2 Biophysical characterization of ACE2‐Fc and ACE2‐(G_4_S) _6_‐Fc

We next proceeded to produce and purify both ACE2‐Fc and ACE2‐(G_4_S)_6_‐Fc. We first analyzed both molecules in Native‐PAGE; the single bands for both confirmed that they existed as monomers, although they ran at an apparent molecular weight ~350 kDa (Figure S1c) instead of the expected theoretical 194 kDa. We next analyzed their molecular weight using size exclusion chromatography/multi‐angle light scattering (SEC/MALS). We found ACE2‐Fc and ACE2‐(G_4_S)_6_‐Fc possessed MW of ~208 kDa and ~212 kDa, respectively, in good agreement with their theoretical size (Figure S1e). Both molecules were predominantly found in the monomeric form: ACE2‐Fc and ACE2‐(G_4_S)_6_‐Fc were ~85% and ~91% monomer after simple Protein A purification, respectively, with the remaining fraction corresponding to oligomers of the proteins and aggregates. We consistently obtained greater yields with ACE2‐(G_4_S)_6_‐Fc production, with an average yield of ~86 mg per 500 mL of transiently transfected culture, which is more than double our typical yield with ACE2‐Fc under identical conditions (~36 mg of protein per 500 mL of culture; Figure S1d).

Finally, we assessed the stability of the molecules using differential scanning calorimetry. The melting temperature *T*_M_ for ACE2‐(G_4_S)_6_‐Fc was ~52 ± 0.6°C (Figure S3). This result is comparable to previously reported values for the *T*_M_ of ACE2 decoys: ~52°C for truncated ACE2‐Fc (residues 18–640) and ~55°C for full length ACE2‐Fc with the collectrin domain (residues 18–740).^8^

### 2.3 Binding of ACE2 decoys to SARS‐CoV‐2 spike proteins

**FIGURE 2:**
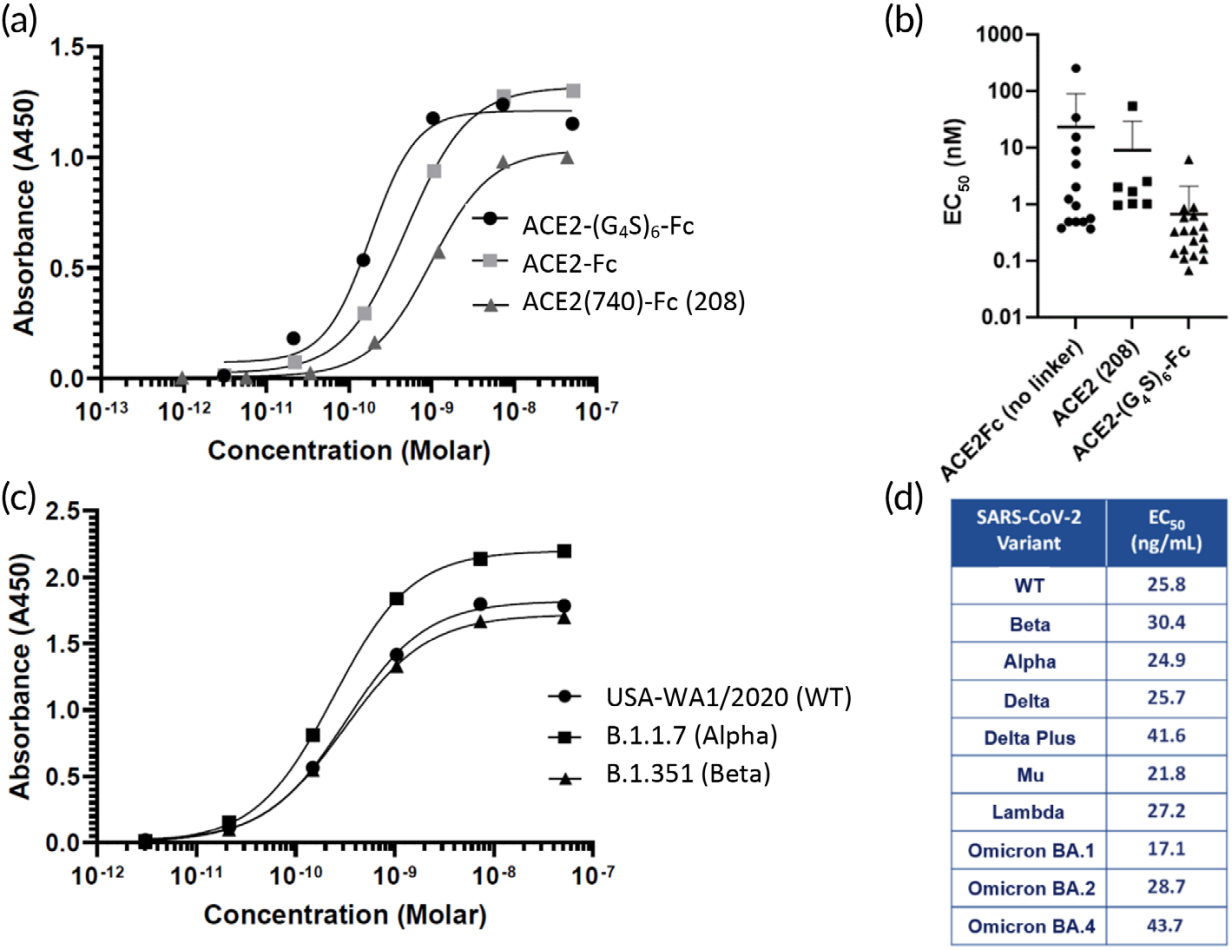
Binding affinity of different ACE2 decoys evaluated by SARS‐CoV‐2 S‐protein ELISAs. (a) Representative concentration‐dependent binding curves for ACE2‐(G_4_S)_6_‐Fc (black circle), ACE2‐Fc (light gray square), and full length ACE2 decoy ACE2(740)‐Fc (gray triangle). (b) ELISA‐derived EC_50_ values for different types of ACE2 constructs. (c) Representative concentration‐dependent binding curves for ACE2‐(G_4_S)_6_‐Fc against S proteins derived from different strains of virus, including WT (USA‐WA1/2020), alpha (B.1.1.7), and beta (B.1.351) strains. (d) Calculated EC_50_s of ACE2‐(G_4_S)_6_‐Fc against variants of interest on ELISA. The ACE2‐(G_4_S)_6_‐Fc drug substance used for the ELISA experiments summarized in panel (d) were produced using stable CHO cell lines being advanced for GMP development. ACE2, angiotensin‐converting enzyme 2; CHO, Chinese hamster ovary; SARS‐CoV‐2, severe acute respiratory syndrome coronavirus 2.

To assess the potency of different ACE2 decoys, we first measured their binding affinity to the spike protein of WT strain USA‐WA1/2020 using ELISA. In addition to ACE2‐Fc and ACE2‐(G_4_S)_6_‐Fc, we also tested full length ACE2‐decoy (i.e., ACE2(740)‐Fc, abbreviated as 208 following the notation in Reference 8). Among the three ACE2 decoys, ACE2‐(G_4_S)_6_‐Fc consistently displayed the highest binding affinity (Figure 2a). Across multiple independently produced batches, ACE2‐(G_4_S)_6_‐Fc consistently exhibited picomolar EC_50_ (mean: 490 pM, or 96 ng/mL; Figure 2b); the median EC_50_ with the most potent batch of ACE2‐(G_4_S)_6_‐Fc was as low as 136 pM, or 27 ng/mL. In contrast, the mean EC_50_ with ACE2‐Fc and ACE2(740)‐Fc, at 3.6 nM (680 ng/mL) and 1.6 nM (370 ng/mL), was ~7.3‐fold and ~3.3‐fold worse than ACE2‐(G_4_S)_6_‐Fc.

The key advantage of ACE2 decoys over conventional monoclonal antibodies (mAbs) is the ability to bind any SARS‐CoV‐2 variant. To confirm ACE2‐(G_4_S)_6_‐Fc can indeed bind different SARS‐CoV‐2 variants, we also measured initially its binding affinity to B.1.1.7 (UK) and B.1.351 (SA) spike proteins using ELISA (Figure 2c), and, later, against more recent variants including the Omicron family (Figure 2d). The binding affinity of ACE2‐(G_4_S)_6_‐Fc to all tested variants was highly comparable (Figure 2d). The ACE2‐(G_4_S)_6_‐Fc evaluated in Figure 2d for breadth of binding across variants of interest was produced using stable CHO pools that have since been advanced into cGMP development. As a control, we also evaluated the binding of RGN10933, a mAb developed by Regeneron that is part of the REGN‐COV2 mAb cocktail that originally received EUA from the FDA prior to the rise of evasive variants. While RGN10933 bound WT and alpha variant S proteins with comparable binding affinity in our ELISA assay (~0.3 ± 0.04 ng/mL and ~0.3 ± 0.08 ng/mL for WT and alpha variants, respectively), we were unable to detect binding against the beta variant, in line with prior reports.^16^ These results underscore the utility of ACE2‐(G_4_S)_6_‐Fc across all SARS‐CoV‐2 variants.

### 2.4 Neutralization potencies of ACE2 decoys in pseudovirus infection assays and against live virus

**FIGURE 3:**
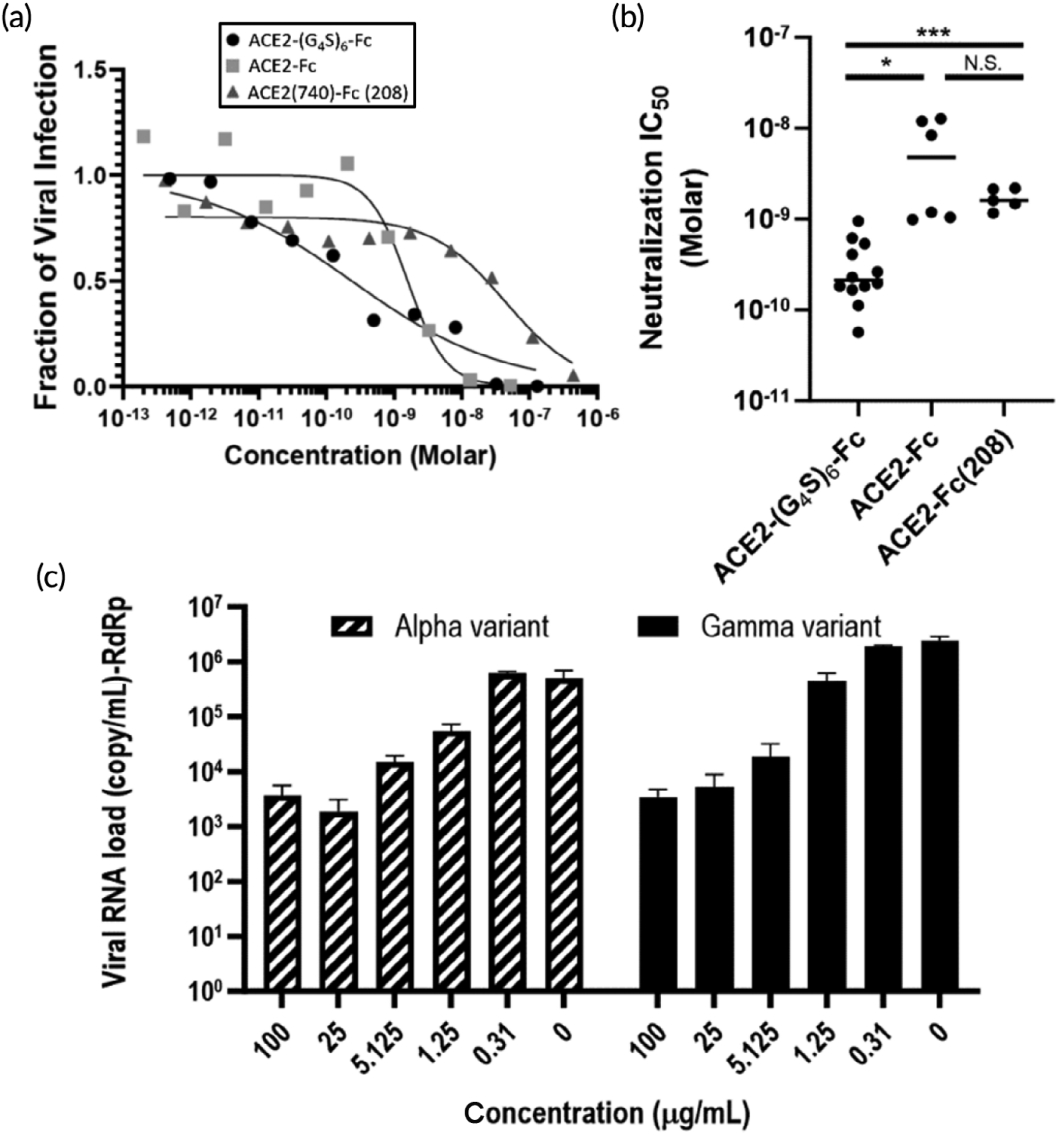
Neutralization potency of different ACE2 decoys against pseudovirus and live SARS‐CoV‐2 virus. (a) Representative infectivity curves of pseudotyped SARS‐CoV‐2 virus across different concentrations of ACE2 decoys. (b) IC_50_ values estimated from the binding curves. Each data point represents independent experiments. Student's *t*‐test was used for comparisons across molecules. (c) Neutralization of live SARS‐CoV‐2 virus. Vero E6 cells were infected 48 h prior at MOI of 0.01 before treatment with ACE2‐(G_4_S)_6_‐Fc. RdRp stands for the RNA‐dependent RNA polymerase, which was the gene target of the RT‐PCR used in this experiment. IC_50_ for Alpha and Gamma variants of 0.96 μg/mL and 0.57 μg/mL, respectively. ACE2, angiotensin‐converting enzyme 2; SARS‐CoV‐2, severe acute respiratory syndrome coronavirus 2. N.S., *, and *** represent “non‐significant”, *p* < 0.05, and *p* < 0.001, respectively.

We next sought to determine whether the increased apparent binding affinity of ACE2‐(G_4_S)_6_‐Fc correlates with greater neutralizing activity. We measured the neutralization potencies of ACE2‐(G_4_S)_6_‐Fc, ACE2‐Fc, and ACE2(740)‐Fc via standard pseudovirus assay, where HEK cells overexpressing ACE2 is infected with lentivirus encoding eGFP transgene pseudotyped with D614G variant of SARS‐CoV‐2 spike protein. The infectivity of the pseudovirus at different ACE2‐decoy concentrations was determined by measuring eGFP fluorescence of cells incubated with varying amounts of ACE2 decoys using flow cytometry. We found ACE2‐(G_4_S)_6_‐Fc neutralized the SARS‐CoV‐2 pseudovirus with picomolar affinity, with an average IC_50_ of 52 ng/mL (Figure 3a,b). In contrast, the neutralization potency of ACE2‐Fc and ACE2(740)‐Fc was nearly 5‐fold and 6‐fold reduced, with IC_50_ ~ 240 ng/mL and ~310 ng/mL, respectively. ACE2‐(G_4_S)_6_‐Fc also possessed ~2‐fold greater IC_90_ than ACE2‐Fc (~2.3 vs. ~4.2 μg/mL). These results confirmed ACE2‐(G_4_S)_6_‐Fc indeed possess greater binding and affinity and neutralization potency than conventional ACE2 decoys.

We proceeded to evaluate the neutralization potency of ACE2‐(G_4_S)_6_‐Fc against live SARS‐CoV‐2 virus. Viral RNA levels in Vero‐E6 cells infected with different live SARS‐CoV‐2 strains were considerably reduced at concentrations in the sub μg/mL range, and the neutralization was even more potent for B.1.1.7 (IC_50_ ~ 120 ng/mL) and B.1.1.28 (IC_50_ ~ 570 ng/mL) mutants (Figure 3c), consistent with the reported improved binding of ACE2 by these mutant spikes. These results both underscore the neutralizing potencies of ACE2‐(G_4_S)_6_‐Fc, and are also consistent with the ability of wildtype ACE2 decoys in neutralizing diverse SARS‐CoV‐2 variants.

### 2.5 ACE2‐(G_4_S) _6_‐Fc effectively traps SARS‐CoV‐2 virus‐like particles (VLPs) in human AM and can be stably nebulized

**FIGURE 4:**
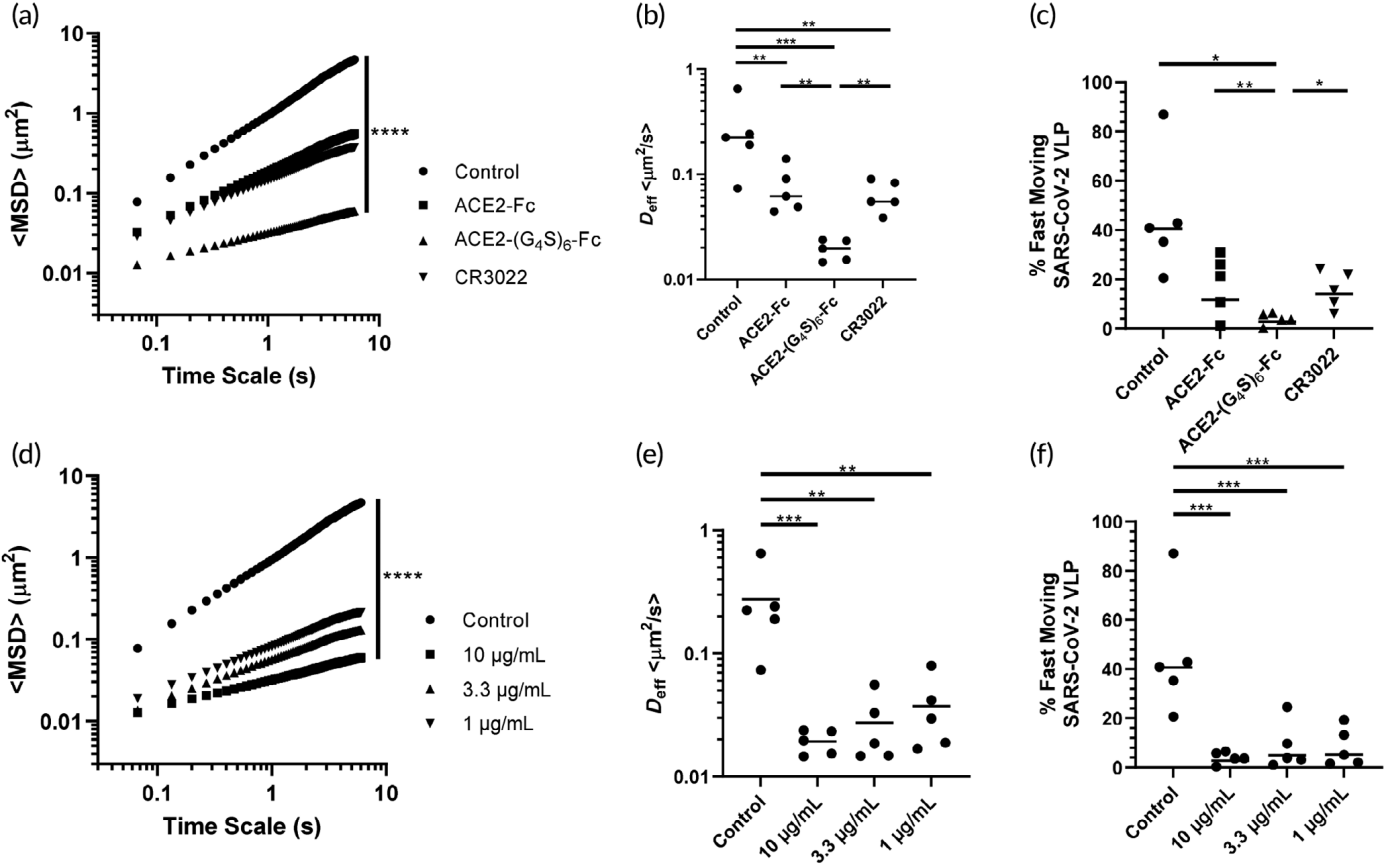
ACE2‐(G_4_S)_6_‐Fc more effectively traps SARS‐2 VLP in human AM than ACE2‐Fc or CR3022, a control anti‐SARS‐CoV‐2 mAb, as measured by high‐resolution multiple particle tracking. (a) Mean squared displacements versus time scale, (b) ensemble averaged effective diffusivities (⟨*D*_eff_⟩), and (c) % fast‐moving SARS‐CoV‐2 VLPs in naïve AM treated with different molecules. (d) Mean squared displacements versus time scale for different concentrations of ACE2‐(G_4_S)_6_‐Fc, (e) ensemble averaged effective diffusivities (*D*_eff_), and (f) % fast‐moving SARS‐CoV‐2 VLPs in naïve AM treated with different concentrations of ACE2‐(G_4_S)_6_‐Fc. MSD and *D*_eff_ were compared across drug constructs or across drug concentrations using one‐sided *t* tests, and unadjusted *p* values are shown as *p < 0.05, **p < 0.01, and ***p < 0.001. ACE2, angiotensin‐converting enzyme 2; AM, airway mucus; MSD, mean squared displacement; SARS‐CoV‐2, severe acute respiratory syndrome coronavirus 2; VLP, virus‐like particle.

To evaluate whether ACE2‐(G_4_S)_6_‐Fc can trap SARS‐CoV‐2 in human AM, we prepared fluorescent SARS‐2 VLP by co‐expressing S protein with GAG‐mCherry fusion construct and visualized the mobility of hundreds to thousands of individual virions in different samples of fresh human AM isolated from extubated endotracheal tubes. These studies were completed before EUA as granted for any COVID vaccines, and thus these specimens likely contained little, if any, endogenous antibodies that could bind and trap SARS‐CoV‐2 VLPs, unlike our previous work with influenza.^26^ Not surprisingly, in the absence of exogenously added antibodies or ACE2‐decoy molecules, SARS‐CoV‐2 VLPs exhibited rapid diffusion in AM, as reflected by the high mean‐squared displacement (MSD) values and the slope of the MSD versus time scale plot (Figure 4a). In contrast, ACE2‐(G_4_S)_6_‐Fc effectively trapped SARS‐2 VLP in AM, as quantified by the markedly lower MSD values and lower slope value. This corresponds to a ~10‐fold reduction in the average ⟨*D*_eff_⟩ of the SARS‐CoV‐2 VLPs compared to naïve AM (Figure 4b), and a nearly 14‐fold reduction in the fast moving viral populations, defined as the fraction of viruses possessing sufficient diffusivity to diffuse across ~50 μm layer in ~1 h) even at just 1 μg/mL conc. in AM (Figure 4c). In contrast, neither ACE2‐Fc nor CR3022, a high‐affinity mAb against S protein, were unable to reduce viral mobility to the same extent as ACE2‐(G_4_S)_6_‐Fc. We then proceeded to assess whether ACE2‐(G_4_S)_6_‐Fc could facilitate muco‐trapping at lower concentrations. We observed a slight dose‐dependent trapping of SARS‐CoV‐2 VLPs, with slightly more effective trapping at higher ACE2‐(G_4_S)_6_‐Fc concentrations, although the difference was not statistically significant (Figure 4d,e). The fraction of fast‐moving SARS‐CoV‐2 VLPs was reduced at all ACE2‐(G_4_S)_6_‐Fc concentrations tested (Figure 4f).

We were interested in investigating whether ACE2‐(G_4_S)_6_‐Fc could similarly trap other ACE2‐targeted virions in AM. Since the causative agent of SARS, SARS‐CoV‐1, also binds to human ACE2 for cellular entry, we next assessed whether ACE2‐(G_4_S)_6_‐Fc could trap SARS‐CoV‐1 VLPs in human AM, and again found ACE2‐(G_4_S)_6_‐Fc mediated potent trapping of SARS‐CoV‐1 VLPs in human AM (Figure S2). This further substantiates the broad value of this decoy approach for any pathogen that may target human ACE2, not just variants of SARS‐CoV‐2.

**FIGURE 5:**
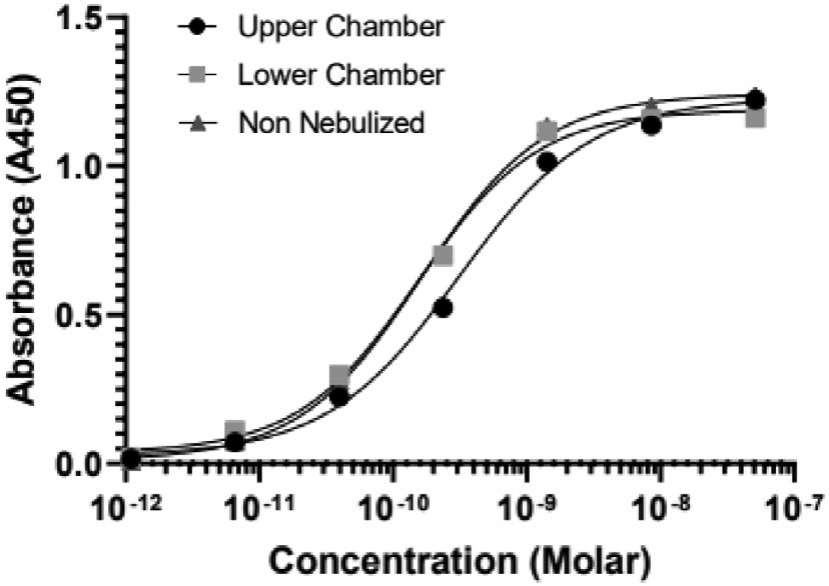
Binding affinity of nebulized ACE2‐(G_4_S)_6_‐Fc evaluated by SARS‐CoV‐2 S‐protein ELISAs. ACE2‐(G_4_S)_6_‐Fc collected from the upper chamber and lower chamber of twin glass impinger are compared to non‐nebulized protein (triangle). ACE2, angiotensin‐converting enzyme 2; SARS‐CoV‐2, severe acute respiratory syndrome coronavirus 2.

The most direct method to achieve therapeutic concentrations of mAb in the respiratory tract, particularly the lung airways, is to directly deliver the mAb via inhalation.^30^ Vibrating mesh nebulizers (VMNs) can nebulize protein therapeutics without generating local heating and shearing that can degrade proteins. We thus tested whether ACE2‐(G_4_S)_6_‐Fc could be stably nebulized using a VMN. Using a Philip's InnoSpire Go VMN, we nebulized ACE2‐(G_4_S)_6_‐Fc, collected the resulting aerosols using a two‐chamber twin glass impinger setup designed to capture aerosols >6.4 μm (upper chamber) and <6.4 μm (lower chamber) following European Pharmacopeia 5.0,^31^ and measured the binding affinity of the recovered nebulized ACE2‐(G_4_S)_6_‐Fc via S‐protein ELISA. We did not observe any appreciable loss in binding affinity of ACE2‐(G_4_S)_6_‐Fc recovered from either the upper or lower chamber, compared to ACE2‐(G_4_S)_6_‐Fc that was not nebulized (Figure 5). Native PAGE also confirmed there was no separation of the heavy chains or detectible aggregation (Figure S3) and differential scanning fluorimetry showed a peak on first derivative ratio around 54°C. These results underscore our ability to produce and stably nebulize ACE2‐(G_4_S)_6_‐Fc for direct inhalation delivery into the respiratory tract.

### 2.6 Intranasal delivery of ACE2‐(G_4_S) _6_‐Fc reduces viral load in the nasal turbinates of hamsters infected with SARS‐CoV‐2

**FIGURE 6:**
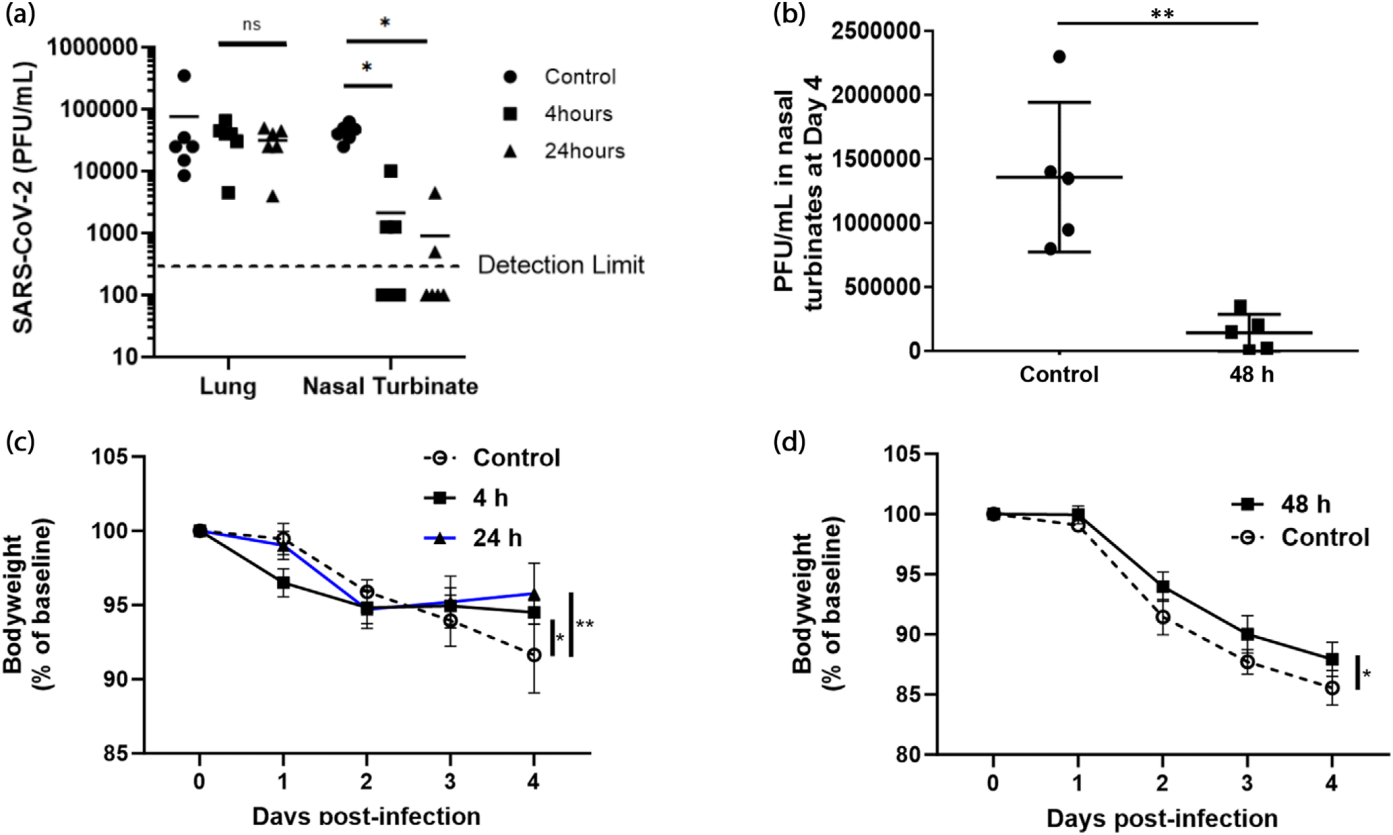
Efficacy of topical ACE2‐(G_4_S)_6_‐Fc treatment in a hamster model of COVID‐19. (a) Viral load in the lung and nasal turbinate tissues of SARS‐CoV‐2‐infected hamsters collected at 4 days post infection, groups treated either 4 or 24 h after infection. (b) Viral load in the nasal turbinates in animals that were withheld treatment until 48 h post infection. (c) Change in bodyweight over the course of the study in animals treated at 4 h or 24 h post infection, compared to control. (d) Change in bodyweight over the course of the study in animals treated at 48 h post infection versus control. PFU/mL or bodyweight were compared across treatment groups using one‐sided *t* tests, and unadjusted *p* values are shown as *p < 0.05, **p < 0.01.

As an in vivo proof‐of‐concept, we conducted a pilot study assessing the efficacy of intranasal delivery of ACE2‐(G_4_S)_6_‐Fc in golden Syrian Hamsters infected with live SARS‐CoV‐2. SARS‐CoV‐2‐infected hamsters develop clinical signs of weight loss, lethargy, hunched back posture, ruffled fur, and rapid breathing, and histopathological changes with high viral loads in the upper and lower respiratory tract, making them a suitable model for testing mAb‐based approaches despite differences in anatomy of the respiratory tract.^32^, ^33^ Most prior studies evaluated mAb against SARS‐CoV‐2 to test the administration of the therapeutic at just 2–6 h following infection.^34^, ^35^, ^36^ Here, we first evaluated initiating daily intranasal dosing of ACE2‐(G_4_S)_6_‐Fc starting at 4 and 24 h postinfection. While we did not observe significant reduction of the viral titers in the deep lung, we saw highly impressive reduction of the viral titers in the nasal turbinates (Figure 6). The majority of the animals receiving treatment starting at 4 and 24 h postinfection had viral titers reduced to below the detection threshold, and the mean reduction ranged from ~40‐fold to ~20‐fold (*p* < 0.05), respectively (Figure 6a). The treated animals also exhibited substantially less weight loss compared to saline control.

Building off these results, we further attempted initiating treatment at 48 h postinfection. Surprisingly, despite this very late initiation of therapy, ACE2‐(G_4_S)_6_‐Fc treatment still provided a ~10‐fold reduction in viral load in the nasal turbinate tissues at the 96 h postinfection time point (Figure 6b). The reduction in viral titers again translated to substantial protection against weight loss over the 2‐day period (*p* = 0.03).

### 2.7 Production of ACE2‐(G_4_S) _6_‐Fc in cGMP‐compliant Chinese hamster ovary (CHO) cells

CHO cells represent the gold standard of mAb manufacturing. To prepare for IND‐enabling preclinical studies, we generated and selected stable CHO cell lines that produced ACE2‐(G_4_S)_6_‐Fc. Encouragingly, we are able to produce ACE2‐(G_4_S)_6_‐Fc with >2 g/L productivity, generated research cell bank clones, and selected a clone to advance toward master cell banking for GMP production. The measured EC_50_ were consistently ~25–30 ng/mL against S‐proteins from various SARS‐CoV‐2 variants (Figure 2d). These binding affinities are comparable to a number of mAbs that have demonstrated clinical utility against sensitive SARS‐CoV‐2 strains.

## 3 DISCUSSION

As part of the unprecedented global effort to develop effective antiviral therapies against SARS‐CoV‐2, there were multiple neutralizing mAbs that were quickly advanced into clinical development. Indeed, mAbs were the first class of therapeutics approved as antivirals for COVID‐19, and the fact that every one of the mAbs advanced into Phase 2/3 studies consistently saw strong efficacy underscores the overall effectiveness of antiviral mAbs. Indeed, when administered early in the course of infection, the antiviral mAbs were generally highly effective (~70%–85%) at reducing risk of hospitalization and death.^37^, ^38^ Despite in many cases the use of mAb cocktails targeting distinct structural epitopes (e.g., REGN‐COV2, Evusheld), every one of the mAbs that had received EUA early on were eventually pulled from the market due to loss of activity against emerging immune evasive variants, particularly Omicron.^39^, ^40^, ^41^, ^42^ This underscores the limited effectiveness of a mAb cocktail approach in preventing viral escape against SARS‐CoV‐2.

Given the concerns about escape mutants, it would be highly advantageous to develop a single mAb‐based product that is not at risk of viral escape. Furthermore, given that there are already three human coronaviruses that target ACE2 as the primary host entry receptor (SARS‐CoV‐2, SARS‐CoV‐1, and HCoV‐NL63), we consider it quite plausible that another ACE2‐targeting respiratory virus will emerge with pandemic potential. Soluble ACE2 decoys can block infections by both SARS‐CoV‐1^43^ and SARS‐CoV‐2.^9^, ^44^ For these reasons, we, along with a number of other groups, became interested in developing ACE2 decoys, with the goal of enabling a universal immunotherapy against all ACE2‐targeted viruses. Contrary to the strategy of engineering ACE2 mutants for improved affinity to S protein, we made a concerted approach to utilize only the WT ACE2 fragment, thus eliminating the potential risk of viral escape mutants that bind WT ACE2 but cannot be captured by the ACE2 mutant. We also believe maintaining Fc effector function to be critical to the therapeutic efficacy of antiviral mAbs, and thus retained the wildtype IgG1‐Fc. These considerations led us to (i) eliminate the collectrin domain on ACE2, (ii) optimize the linkage between the extracellular fragment of ACE2 with the IgG1‐Fc, and (iii) incorporate a “muco‐trapping” Fc. We found that ACE2‐(G_4_S)_6_‐Fc, engineered with these features, not only was able to achieve exceptionally consistent binding to all variants of SARS‐CoV‐2, but also could be stably nebulized and trap SARS‐CoV‐2 VLPs in human AM. These features likely contributed to the effective suppression of viral load in nasal turbinates and clinical signs in hamsters in this study, even when dosed as late as 48 h postinfection. These results strongly substantiate the promise for advancing ACE2‐(G_4_S)_6_‐Fc into clinical development; we are currently undertaking the necessary preclinical activities and GMP manufacturing to do so.

Early work with ACE2 decoys against SARS‐CoV‐2 infection showed that simply linking ACE2 to an Fc domain inhibited infection in vitro with a modest EC_50_ of ~24 μg/mL, while retaining effector function of the Fc.^13^ This finding motivated investigators to pursue strategies to further improve ACE2 decoys. One common approach is to conduct directed evolution to introduce targeted mutations to recombinant ACE2 using structural knowledge and screening of mutant libraries.^5^, ^6^, ^8^ These strategies resulted in engineered ACE2 decoys with EC_50_s to S protein closer to 150 ng/mL.^45^ One study tested an inhaled engineered ACE2 decoy in a K18‐hACE2 transgenic mouse model of COVID‐19, which prolonged survival and reduced viral load consistent with our findings in hamsters in this work.^46^ In contrast with using targeted mutations to increase affinity of ACE2 decoy molecules, other groups have sought to improve affinity by increasing ACE2 valency, ranging from dimeric ACE2‐Fc (fusion of ACE2 to IgG1‐ or IgG4‐Fc), trimeric ACE2,^15^, ^47^ tetrameric ACE2,^12^ to hexameric constructs via the oligomerization motif from an IgM Fc domain to create hexavalent ACE2‐based molecules,^48^ or via incorporating an IgM tailpiece (PTLYNVSLVMSDTAGTCY) that enable pentamerization or hexamerization of IgGs.^17^ Indeed, trimerization of Fc‐null ACE2 decoys by fusing ACE2 to T4 foldon or helix bundle through flexible linkers increased the affinity of the molecule to SARS‐CoV‐2 S protein by 5‐ to 10‐fold compared to the monomer, and shifted the IC_50_ from >7500 ng/mL to hundreds of ng/mL. One report of an IgM‐like inhalable ACE2 fusion protein was shown to neutralize SARS‐CoV‐2 variants with an IC_50_ of ~13–131 ng/mL.^17^ In another study, hexavalent ACE2‐based molecules were demonstrated an IC_50_ of 0.18 ± 0.04 nM.^48^ The potencies achieved herein with ACE2‐(G_4_S)_6_‐Fc are comparable to those previously observed with the trimeric ACE2 and the “IgM” versions of ACE2 presentation on a mass concentration basis. While our strategy is more akin to the multimerization approach, it differs in that we believe two ACE2 fragments per molecule would confer sufficient binding avidity for the molecule to be highly active at concentrations we can readily achieve physiologically, if we could extend the reach of the two ACE2 fragments. Indeed, by utilizing a longer extended linker, ACE2‐(G_4_S)_6_‐Fc achieved binding affinity and neutralization potencies comparable to many of the affinity‐matured or highly multimerized ACE2 decoys, and conferred effective protection of the upper respiratory tract in vivo. Finally, we are also unique in our focus to engineering molecules with a “muco‐trapping” Fc.

Intranasal delivery of ACE2‐(G_4_S)_6_‐Fc conferred highly effective suppression of SARS‐CoV‐2 in the nasal turbinates of hamsters, the key site for virus replication and dissemination, even when treatment was withheld until 48 h after infection. Notably, prior studies in the same hamster model have shown that intravenously administered mAbs were unable to effectively reduce viral load in the nasal turbinates of hamsters, despite some protection in the lower airways.^33^ We believe the lower respiratory tracts in rodents, particularly the deep lung, have greater blood permeability than the upper airways; this enhances the relative basal‐to‐apical mAb distribution, which in turn enabled modest protection. In contrast, following intranasal delivery, we were likely achieving and sustaining highly inhibitory concentrations of ACE2‐(G_4_S)_6_‐Fc in the nasal turbinates, which reduces the viral load locally. These results directly demonstrate that topical treatment of a mAb‐based antiviral could afford meaningful antiviral activity.

Due to stark anatomical differences in the respiratory tract compared to humans, hamster is typically not used for inhalation studies; instead, models like dogs are frequently utilized. Unfortunately, we were limited to the fact that hamster was the only available animal model for SARS‐CoV‐2 infection at the time, and to this day represents the gold standard utilized by nearly all candidate therapies for COVID‐19. As a nebulization setup was not available in the BSL‐3 facility, we could not actually evaluate direct nebulized delivery of our molecule into the lungs of the animals. This limited us to administering our treatments by intranasal dosing. While adequate for delivery to the nasal turbinates, intranasal administration notoriously results in spotty distribution within the lungs of rodents. Thus, we consider the limited anti‐viral effects observed in the lungs of hamsters in this work is likely an artifact from non‐uniform pulmonary distribution following intranasal instillation of drug, a notorious challenge with intranasal delivery to rodents. Such non‐uniform delivery will likely be resolved by the use of VMNs in human studies, which offers control of delivery throughout the entire respiratory tract.

ACE2 preferentially traffics to the apical membrane of airway epithelium; this is consistent with the apical infection and apical shedding observed with SARS‐CoV‐1^20^ and SARS‐CoV‐2.^19^ This pathophysiology also means that viruses are concentrated either within the luminal airway epithelium or in the AM directly overlaying the cells. In turn, they are difficult to reach by systemically‐dosed antivirals, particularly biologics. Indeed, antiviral mAbs take days to reach Cmax locally in the respiratory tract, and typically far less than 1% of the injected dose actually does so. For instance, a Phase 2 study of the anti‐influenza mAb CR6261 showed that Cmax in the airways only occurred on Days 2–3 post‐dosing, with Cmax in the nasal fluid well below the IC_50_ of the mAb.^49^ In contrast, direct delivery into the respiratory tract by inhalation mitigates these biodistribution limitations. Inhalation achieves highly inhibitory concentrations of the drug virtually instantaneously, which is expected to maximize efficacy and increase the time window for initiating treatment, while also minimizing the dose needed. While jet nebulizers and ultrasonic nebulizers generate sufficient heat and shearing to denature proteins, the latest generations of VMN can support stable nebulization of various biologics as long as the formulation is suitably optimized. Indeed, we recently demonstrated stable nebulization of a potent muco‐trapping mAb (IN‐006) against SARS‐CoV‐2 using VMN that met regulatory requirements for initiating clinical studies,^50^ and completed a Phase 1 trial demonstrating the safety, tolerability, and surprisingly prolonged pulmonary PK in healthy adults.^51^ Given that ACE2‐(G_4_S)_6_‐Fc can be stably nebulized as IN‐006, we anticipate a similar once per day inhaled dosing regimen would similarly sustain highly inhibitory levels of ACE2‐(G_4_S)_6_‐Fc in both the upper and lower respiratory tracts in humans, much greater than what could be achieved via systemic delivery.

Finally, mucus is often overlooked in the context of immune protection against foreign pathogens. This is not surprising as IgG has long been thought to mediate protection systemically by interacting with the immune cells and complement proteins. Nonetheless, there are abundant quantities of IgG naturally present in human AM at levels that are comparable to sIgA, implicating a potential role for IgG in mucosal protection of the respiratory tract. The notion that IgG can work in tandem with mucins to immobilize pathogens in mucus was only demonstrated less than a decade ago by us, first with engineering mAbs that could immobilize Herpes Simplex Virus in cervicovaginal mucus leading to sterilizing immunity against vaginal Herpes transmission,^27^, ^52^ and more recently in engineering antibodies that can effectively trap SARS‐CoV‐2^53^ and Ebola VLPs^24^ in fresh human AM. Thus, IgG's potential muco‐trapping effector function is generally overlooked in the development of antiviral mAb interventions. Nonetheless, muco‐trapping offers a number of highly desirable features, including directly intervening in the viral life cycle by immediately halting the spread of infection, unlike small molecule antivirals that fail to inhibit already shed progeny virus. Furthermore, by harnessing natural mucociliary and/or cough‐driven AM clearance, muco‐trapping mAbs can directly remove potentially inflammatory viral antigens from the respiratory tract,^26^, ^27^, ^28^, ^52^, ^54^, ^55^, ^56^ thus attenuating the pneumonia and inflammation that results in severe COVID. Interestingly, higher affinity between IgG‐Fc and mucins does not enhance muco‐trapping potencies; instead, there is an optimal weak affinity supported by specific Fc glycosylation that enables IgG to quickly diffuse through mucus and accumulate on the surface of the virus, while enabling effective trapping of mAb/virion complexes.^10^, ^11^ Here, we produced our ACE2 immunodecoy using specific mammalian cell lines that we previously found to generate Fc N‐glycosylation supporting the muco‐trapping effector function. Such N‐glycosylations would likely be absent from molecules generated from bacterial and fungal systems. Finally, combined with the outstanding safety of biologics, we anticipate inhaled “muco‐trapping” mAbs and immunodecoys will eventually become a broadly adopted approach as early therapy for both mild and moderate COVID‐19 and other acute respiratory viral infections.

## 4 MATERIALS AND METHODS

### 4.1 Cloning of ACE2‐Fc and ACE2‐(G_4_S) _6_‐Fc

Plasmid containing ACE2 without CD domain fused to monomeric Fc domain (pAce2‐mFc) and SARS‐CoV‐1 S protein were kind gifts from Jason's McLellan's lab. Double stranded DNA strings, gblocks®, containing (GGGGS)_6_‐Fc fusion were purchased from IDT DNA. To generate the plasmid for ACE2‐(G_4_S)_6_‐Fc (pAce2‐LdFc), pAce2‐mFc was digested with BamH and XhoI, and (GGGGS)_6_‐Fc was inserted by Gibson assembly. The reaction was transformed to chemically competent TOP10® (Thermo Fisher) and plated in LB + carbenicillin plates. Sanger sequencing was used to confirm assembly. To generate the plasmid for ACE2‐Fc (pAce2‐dFc), Fc was amplified from (GGGGS)_6_‐Fc gblock® using primers pF1 and pR1 using high‐fidelity Phusion polymerase. The PCR product was then cloned into pAce2‐mFc digested with BamH and XhoI by Gibson assembly, as previously described. Plasmid for soluble expression of ACE2 (740)‐Fc (CVD‐208^8^) was a kind gift from James Wells lab.

### 4.2 Cloning of SARS‐CoV‐2 wild type and mutant S proteins for soluble expression

The plasmid nCov‐2.sol, which encodes SARS‐CoV‐2 wild type S protein with 2P mutations,^57^ a mutated furin site, a C‐terminal foldon, and hexa‐histidine tag was a kind gift from Jason McLellan's lab.^58^ It is used for soluble expression of S protein. To generate S proteins encoding the mutations in the South African strain of SARS‐CoV‐2 beta variant in nCov2.sol, the plasmid was digested with AgeI and NheI. PCR primers Pf2, Pr2, Pf3, and pR3 were designed to amplify 2 fragments from S protein with mutations K417N, E484K, and N501Y. These two fragments were cloned into digested nCov2.sol by Gibson assembly to generate a full‐length S protein with beta variant mutations. Proper assembly of the protein was confirmed by Sanger sequencing (Genewiz). Hexapro mutations^59^ intended to stabilize the soluble protein were inserted into wild type and SA‐nCov2.sol by amplifying nCov2.sol with primers Pf5 and Pr5, which amplify the vector and S protein 1–816 and 943–1208, and inserting a DNA fragment encoding S protein residues 817–942 with hexa‐pro mutations by Gibson assembly. The resulting vectors are henceforth referred to as WT‐hexapro‐nCov2.sol and SA‐hexapro‐nCov2.sol. The fragment with hexa‐pro mutations was amplified from plasmid UK‐hexapro‐nCoV2.xdna. This plasmid encodes for the S protein of the alpha variant with hexa‐pro mutations and was purchased from Twist Bioscience.

### 4.3 Cloning of SARS‐CoV‐2 wild type and mutant S proteins for lentivirus production

Plasmids needed for the generation of SARS‐CoV‐2 pseudotyped infectious lentivirus were generated as follows. The plasmid pUC57‐2019‐nCoV‐S containing human codon optimized Spike DNA was purchased from Genscript Molecular Cloud. This DNA was amplified using primers to generate a C‐terminal truncation^43^, and cloned into the mammalian expression vector pAH to generate pAH‐S‐CoV‐2‐ΔCt. To generate pAH‐S‐Cov2‐ΔCt with beta variant mutations, pAH‐SA‐CoV‐2‐ΔCt.vlp, one fragment of beta variant S protein was amplified from SA‐nCov1.sol using primers Pf7 and Pr7. Another fragment of WT S protein was amplified with Pf8 and Pr8 from WT pAH‐S‐CoV‐2‐ΔCt. The fragments were then cloned into pAH cloning vector digested with KpnI and XhoI by Gibson assembly. Lentivirus was made using a third generation packaging system using four plasmids pMDLg/pRRE (Addgene) + pRSV‐Rev (Addgene) + pAH‐S‐CoV‐2‐ΔCt and a transfer plasmid (pLL7.0 EGFP) containing EGFPgene which was used to track infection. Plasmids needed for generation of non‐replicating SARS‐Cov‐2 wild type and SA VLPs were generated as follows. A gblock® encoding the C‐terminal domain of SARS‐CoV‐1 was purchased from IDT DNA. WT‐hexapro‐nCov2.sol, SA‐hexapro‐nCov2.sol, and UK‐hexapro‐nCov2.sol were digested with BamHI and XhoI to remove the foldon domain and his tag, and then the gblock® containing the Cterm of SARS‐CoV‐1 was introduced by Gibson assembly.

### 4.4 Production and purification of ACE2‐LFc and ACE2‐dFc

Endotoxin free pAce2‐dFc and pAce2‐LdFc for transfections were purified using NucleoBond Xtra Midi Plus EF kit (Macherey‐Nagel). ACE2‐Fc and ACE2‐(G_4_S)_6_‐Fc were produced in Expi293T™ cells (sex of cell line: female) by transient transfection using ExpiFectamine™ Transfection Kit (Thermo Fisher). Five hundred milliliters of cultures were used, and cells were harvested before viability dropped below ~75%. Cell culture supernatants were concentrated using tangential flow (Sartorius Vivaflow 50 crossflow cassette system with 100,000 MWCO cassette with polyethersulfone membrane) for purification by protein A chromatography. Three 5 mL HiTrap Protein A columns (Cytivia) were connected in tandem to a NGC Quest 10 FPLC (BioRad). The columns were equilibrated with five column volumes (CV) of 10 mM sodium phosphate buffer, pH 7.0. Protein was loaded into the column at 0.5 mL/min, followed by a 10 CV wash step with phosphate buffer, and subsequently eluted by a 5 CV isotonic elution step with 100% 0.2 M glycine buffer pH 2.0; 3 mL fractions were collected in tubes filled with 300 μL of 1 M Tris buffer with 0.2% polysorbate 80, pH 8. Purity of each fraction was assessed by SDS‐PAGE, and the fractions with no extra bands were combined and buffer exchanged into 20 mM His, mg/mL sucrose, 0.2% polysorbate 80, 130 mM NaCl, pH 6.2 (standard buffer) using Spin‐X® UF 50k MWCO PES spin columns (Corning). Following buffer exchange, the proteins were filtered with a 0.22 μm filter and flash frozen in liquid nitrogen before been stored at −80°C.

### 4.5 Production and purification of recombinant SARS‐CoV‐2 S proteins

Endotoxin free ncov2.sol, WT‐hexapro‐nCov2.sol, SA‐hexapro‐nCov2.sol, and UK‐hexapro‐nCov2.sol were purified using NucleoBond Xtra Midi Plus EF kit. The plasmids were transfected into Expi293T™ using ExpiFectamine™ Transfection Kit. Five hundred milliliters of cultures were used, and cells were harvested before viability dropped below ~45%. Cell culture supernatants were concentrated 10‐fold by tangential flow (Sartorius Vivaflow 50 crossflow cassette system with 100,000 MWCO cassette with polyethersulfone membrane). The concentrated supernatant was incubated with 1 mL of Ni‐Nta agarose resin (Qiagen) overnight before being recovered with a gravity‐flow column (Bio‐Rad). The resin was then washed with several column volumes of PBS with 20 mM imidazole, followed by elution with PBS with 500 mM imidazole. The proteins were then buffer exchanged into PBS or 20 mM Tris with 120 mM sucrose and 20 mM sodium chloride pH 7 using Spin‐X® UF 50k MWCO PES spin columns. Following buffer exchange, the proteins in Tris‐sucrose buffer were flash frozen in liquid nitrogen before been stored at −80°C.

### 4.6 Production of VLPs

Fluorescent VLPs were made by cotransfection of pGAG‐mcherry plasmid (kind gift from Gummuluru lab) and CoV‐2 S protein plasmid in a 1:1 ratio. Nonreplicating lentivirus pseudotyped with SARS‐CoV‐2 alpha (B.1.1.7) spike protein were created using the following plasmids, in a 1:1:1:2 ratio: pMDLg/pRRE, pRSV‐REV, SARS‐CoV‐2 alpha spike, and pLL7 GFP. Nonreplicating lentivirus pseudotyped with SARS‐CoV‐2 beta spike (B.1.351) were created using the same plasmids/ratio above with SARS Cov2 alpha spike replaced with SARS Cov2 beta spike. All plasmids were purified using NucleoBond Xtra Midi Plus EF kit. The plasmids were transfected into LVMaxx using the LVMaxx Transfection kit. Each VLP was made in 60 mL cultures, and harvested after 48 h. The VLPs were purified using 25% sucrose (in 25 mM HEPES/130 mM NaCl) cushion spin protocol. Three milliliters of 25% sucrose solution was add to each Beckman Coulter ultracentrifuge tube, which then had 7 mL of cell culture supernatant gently layered on top. The tubes were then spun at 36,000 rpm for 2.5 h at 4°C. The sucrose/supernatant was then aspirated off, and 20 μL of 10% sucrose solution was placed on top of the VLP pellet. After 24 h at 4°C, the VLPs were then aliquoted and stored at −80°C.

### 4.7 3D models

UCSF Chimera 1.14^60^ was used to generate all the protein models, and UCSF Chimera X 1.1^61^ was used to render the model for publication. ACE2‐Fc and ACE2‐(G_4_S)_6_‐Fc were constructed using models 6M17 for ACE2,^62^ 1HZH for human IgG,^63^ and 1EIB for GGGGS linker.^64^ ACE2‐Fc and ACE2‐(G_4_S)_6_‐Fc bound to S protein were generated by matching the ACE2 of ACE2‐Fc and ACE2‐(G_4_S)_6_‐Fc with the RBD‐bound ACE2 in the “all‐up” S protein model 7A98.^29^ The predicted 3D model of ACE2‐Fc with collectrin domain was modified from reference 65.

### 4.8 SEC‐MALS measurements of purified proteins and native PAGE

Solutions containing 1.0 mg/mL ACE2‐(G_4_S)_6_‐Fc or Ace2‐Fc were prepared in standard buffer. One hundred microliters of these solutions were then loaded into a Superdex 200 Increase 10/300 GL (Cytivia) mounted on a NGC Quest 10 FPLC (BioRad). The column was pre‐equilibrated with PBS, and the whole run was performed at 0.5 mL/min. The molecular weight of proteins eluting from the column was determined using a Mini Dawn multi‐angle light scattering detector and its companion software Astra8 (Wyatt), assuming an extension coefficient of 1.92. Molecular weight was calculated for two independent batches of ACE2‐(G_4_S)_6_‐Fc and two batches of Ace2‐Fc. For native PAGE, 5 μg of protein were loaded onto 3%–12% Bis‐Tris gels (Invitrogen), and the gels were ran as described by the manufacturer's protocol.

### 4.9 Scanning differential fluorimetry

The melting temperature of ACE2‐LFC was determined by nanoDSF using a Promethius NT.48 (Nanotemper Technologies). Samples were heated up from 25°C to 95°C at a rate of 1°C/min. Samples were measured in triplicate. Reported data is the average of three independent repeats.

### 4.10 ELISA

ELISA binding assays were performed using 96‐well half‐area plates (Fisher Scientific, Costar 3690) coated with 0.5 μg/mL of S protein and incubated overnight at 4°C. ELISA plates were blocked the following day with 5% (w/v) milk (LabScientific MSPP‐M0841) with Tween 20 (Fisher Scientific BP337‐100) at a 1:2000 dilution at room temperature for 1 h. Samples were diluted in 1% (w/v) milk with Tween 20 at a 1:10,000 dilution and plated once the blocking had commenced and the 5% milk had been discarded. Samples were incubated at room temperature for 1 h, and the solution was discarded after the 1 h incubation. Plates were then washed with PBS containing Tween 20 at a 1:2000 dilution four times. A peroxidase‐conjugated goat anti‐human IgG Fc antibody (Rockland 709‐1317) was diluted in 1% milk with Tween 20 at a 1:5000 dilution, plated, and incubated at room temperature for 1 h. The solution was then discarded and washed with PBS with Tween 20 two times, followed by washing with just PBS two more times. Plates were developed with TMB solution (ThermoFisher 34029), and development was stopped by adding 2 N HCl (Sigma‐Aldrich 320331). The absorbance at 450 nm and 595 nm was then measured with a microplate photodetector (Fisher Scientific, accuSkan FC).

### 4.11 Neutralization assays

A series of 10 four‐fold serial dilutions were made of ACE2‐Linker‐Fc or ACE2‐Fc or IgG starting at 20 μg/mL in OptiMEM. Ten microliters of each dilution was added to wells of a 96‐well plate in triplicate. To each of these dilutions 0.5 μL of SARS‐CoV‐2 pseudotyped lentivirus (diluted in OptiMEM to 10 μL, MOI 1, titer estimated using infection of HEK293‐ACE2 cells, a female sex cell line) was added and incubated for 30 min at room temperature. Three wells containing 20 μL of OptiMEM and three wells containing 19.5 μL OptiMEM + 0.5 μL pseudovirus serve as controls to normalize and calculate IC_50_. After 30 min 5000 HEK‐ACE2 cells in 100 μL of DMEM + 10% FBS were added to each well of the plate and incubated at 37°C 5% CO_2_ for 72 h. After 72 h the media was removed carefully without disrupting the cells and cells were trypsinized and analyzed by flow cytometry (Attune NxT, ThermoFisher) and EGFP fluorescence was recorded for each well. The MFI for EGFP fluorescence of the triplicate wells was averaged and plotted against concentration of ACE2/mAb and a four‐parameter non‐linear regression was used to estimate IC_50_ of neutralization.

### 4.12 Neutralization activity of HuNAbs against live SARS‐CoV‐2 (adapted from Reference 33)

Neutralization assays against live SARS‐CoV‐2 were conducted using HKU‐001a strain, GenBank accession no: MT230904.1, a clinical isolate,^66^ in a certified biosafety level 3 (BSL‐3) laboratory. Briefly serial dilutions of antibodies were mixed with 50 μL of SARS‐CoV‐2 (1 x 10^3^ plaque forming units/mL, PFU/mL) in 96‐well plates and incubated for 1 h at 37°C. Mixtures were then transferred to 96‐well plates that had been pre‐seeded with 1 x 10^4^/well Vero E6 cells and were then incubated at 37°C. After 48 h, the culture medium was removed and wells were air‐dried in a bio‐safety cabinet for 20 min. Nucleic acid extraction and real‐time RT‐PCR assays for SARS‐CoV‐2 RNA detection were performed as previously described.^67^

### 4.13 Mucus trapping

Multiple particle tracking analysis of fluorescent SARS‐CoV‐2 VLPs in human AM was performed as described in Reference 24. Human AM was obtained from healthy adult patients intubated for general anesthesia during elective surgery (for a non‐pulmonary indication), following a protocol that was deemed nonhuman subjects research by the UNC‐CH IRB. Briefly, solutions of fluorescent VLPs and ACE2‐Fc or ACE2‐(G_4_S)_6_‐Fc were added to ~10 μL of fresh, undiluted AM in custom‐made glass chambers. The samples were then incubated at 37°C for ~30 min before microscopy. PBS and antibody CR3022 (final concentration 10 μg/mL) were used as negative and positive controls, respectively. The same AM was used for all runs to allow direct comparison among samples. Videos of VLPs diffusing in AM were recorded with MetaMorph software (Molecular Devices, Sunnyvale, CA) at a temporal resolution of 66.7 ms. Videos were analyzed using NetTracker from AI Tracking Solutions to convert video raw data to particle traces. Time‐averaged MSDs and effective diffusivity were calculated by transforming particle centroid coordinates and transformed into time MSDs with the formula ⟨Δ*r* ^2^(*τ*)⟩ = [*x*(*t* + *τ*) – *x*(*t*)]^2^ + [*y*(*t* + *τ*) – *y*(*t*)]^2^, where *τ* is the time scale or time lag.

### 4.14 Hamster study

The animal experiments in this study were approved by the University of Hong Kong (HKU) Committee on the Use of Live Animals in Teaching and Research (5193‐19) and conducted in compliance with the ARRIVE guidelines. Randomization of the animals was performed using an online random number generator (https://www.graphpad.com/quickcalcs/randomize1/). Male and female Syrian hamsters, aged 6–10 weeks, were obtained from the Chinese University of Hong Kong Laboratory Animal Service Centre through the HKU Centre for Comparative Medicine Research. Evaluation of ACE2‐(G_4_S)_6_‐Fc was performed in our established golden Syrian hamster model for COVID‐19 as described previously with some modifications.^32^, ^68^, ^69^ Four groups (*n* = 8 per group) were dosed intranasally with ACE2‐(G_4_S)_6_‐Fc either as a prophylactic (4 h before virus challenge) or as therapeutic (4, 24, or 48 h post‐challenge). One group dosed with PBS served as negative control. Each animal was intranasally challenged with 50 μL of 2 × 10^6^ plaque‐forming units/mL of SARS‐CoV‐2 at 0 days postinfection (0 dpi) under intraperitoneal ketamine (200 mg/kg) and xylzine (10 mg/kg) anesthesia. The hamsters were then dosed daily with ACE2‐(G_4_S)_6_‐Fc until they were sacrificed at 4 dpi. Viral load was quantified in nasal turbinate by qRT‐PCR and normalized by β‐actin (internal gene control).

### 4.15 Nebulization study

ACE2‐(G_4_S)_6_‐Fc in standard buffer at 10 mg/mL was nebulized using a Phillips Innospire Go VMN. Aerosols were collected into a glass impinger setup with upper and lower chambers, following protocol guidance in European Pharmacopeia 5.0. The nebulizer was run until it was visually dry. Then, buffer was added to the different chambers of the glass impinger to recover the deposited antibodies. Aggregate formation in the upper chamber, lower chamber, and left‐over (“dead volume”) samples was assessed by SEC using a A EN‐Rich 650 size exclusion column (Bio‐Rad) mounted on a NGC Quest 10 FPLC (BioRad) and native PAGE as described previously. Binding affinity of nebulized molecules were assessed by S‐protein ELISA as described above.

## AUTHOR CONTRIBUTIONS

**Karthik Tiruthani:** Data curation (equal); formal analysis (equal); writing – original draft (equal); writing – review and editing (equal). **Carlos Cruz‐Teran:** Data curation (equal); formal analysis (equal); writing – review and editing (equal). **Jasper Chan:** Data curation (equal); formal analysis (equal); writing – review and editing (equal). **Alice Ma:** Data curation (equal); investigation (equal). **Morgan McSweeney:** Writing – original draft (equal); writing – review and editing (equal). **Whitney Wolf:** Data curation (equal); investigation (equal); writing – review and editing (equal). **Shoufeng Yuan:** Data curation (equal); formal analysis (equal); writing – review and editing (equal). **Vincent Poon:** Data curation (equal); formal analysis (equal); writing – review and editing (equal). **Chris C. S. Chan:** Data curation (equal); formal analysis (equal); writing – review and editing (equal). **Lakshmi Botta:** Data curation (equal); formal analysis (equal); writing – review and editing (equal). **Brian Farrer:** Data curation (equal); formal analysis (equal); writing – review and editing (equal). **Ian Stewart:** Formal analysis (equal); investigation (equal); writing – review and editing (equal). **Alison Schaefer:** Data curation (equal); formal analysis (equal); writing – review and editing (equal). **Jasmine Edelstein:** Data curation (equal); formal analysis (equal); writing – review and editing (equal). **Priya Kumar:** Investigation (equal); writing – review and editing (equal). **Harendra Arora:** Investigation (equal); writing – review and editing (equal). **Jeff Hutchins:** Supervision (equal); writing – review and editing (equal). **Anthony J. Hickey:** Conceptualization (equal); methodology (equal); supervision (equal); writing – review and editing (equal). **Kwok‐Yung Yuen:** Conceptualization (equal); data curation (equal); formal analysis (equal); methodology (equal); supervision (equal); writing – original draft (equal); writing – review and editing (equal). **Samuel K. Lai:** Conceptualization (equal); formal analysis (equal); funding acquisition (equal); investigation (equal); methodology (equal); project administration (equal); resources (equal); supervision (equal); writing – original draft (equal); writing – review and editing (equal).

## CONFLICT OF INTEREST STATEMENT

S.K.L. is founder of Mucommune, LLC and currently serves as its interim CEO. S.K.L. is also founder of Inhalon Biopharma, Inc., and currently serves as its CSO, Board of Director, and Scientific Advisory Board. S.K.L. has equity interests in both Mucommune and Inhalon Biopharma; S.K.L.'s relationships with Mucommune and Inhalon are subject to certain restrictions under University policy. The terms of these arrangements are managed by UNC‐CH in accordance with its conflict‐of‐interest policies. K.T., C.C.‐T., and S.K.L. are inventors on a patent application that has been licensed by Inhalon Biopharma. M.M., L.B., B.F., and J.T.H. are employees of Inhalon Biopharma, and own company stocks. The remaining authors declare no conflicts of interest.

### PEER REVIEW

The peer review history for this article is available at https://www.webofscience.com/api/gateway/wos/peer-review/10.1002/btm2.10650.

## Supporting information


**Figure S1.** Computational prediction of (a) ACE2‐Fc and (b) ACE2‐(G_4_S)_6_‐Fc docked on S protein with three RBD domains in the “up” position. While ACE2‐Fc is expected to only engage one RBD, ACE2‐(G_4_S)_6_‐Fc is capable of engaging two RBDs (depicted in red). (c) Native‐PAGE of ACE2‐Fc (lane 2) and ACE2‐(G_4_S)_6_‐Fc (lane 3). (d) Yield of ACE2‐Fc and ACE2‐(G_4_S)_6_‐Fc after protein A affinity chromatography. Proteins were purified from 500 mL cultures of Expi293T cells. (e) Size exclusion chromatography of ACE2‐(G_4_S)_6_‐Fc and ACE2‐Fc.
**Figure S2.** ACE2‐(G_4_S)_6_‐Fc effectively traps SARS‐CoV‐1 VLP in human AM. (A) Fraction of fast‐moving SARS‐CoV‐1 and (B) ensemble‐averaged effective diffusivities of SARS‐CoV‐1 in AM treated with various mAbs or ACE2 decoys.
**Figure S3.** Biophysical characterization of nebulized ACE2‐(G_4_S)_6_‐Fc. (A) Native‐PAGE of nebulized ACE2‐(G_4_S)_6_‐Fc. Samples were collected from the upper chamber (lanes 2, 5, 8), lower chamber (lanes 3, 6, 9), and left‐over liquid (“dead volume”) after nebulization (lane 4, 7, 10) of the nebulization device. Data is shown for three repeats. (B) Size exclusion chromatography of ACE2‐(G_4_S)_6_‐Fc before nebulization and samples collected from the upper chamber, lower chamber, or left‐over liquid of the nebulization apparatus. Data representative of three repeats is shown.
**Figure S4.** Differential scanning fluorimetry of ACE2‐(G_4_S)_6_‐Fc. Data for three independent repeats is shown in the figure.

## Data Availability

The data that support the findings of this study are available from the corresponding author upon reasonable request.
