# Supplementary material for: Engineering a “muco‐trapping” ACE2‐immunoglobulin hybrid with picomolar affinity as an inhaled, pan‐variant immunotherapy for COVID‐19
Source: Bioeng Transl Med. 2024 Feb 7;9(4):e10650. doi: 10.1002/btm2.10650 (PMC11256170; doi:10.1002/btm2.10650)
Supplement: Supplementary file 1 — Figure S1. Computational prediction of (a) ACE2‐Fc and (b) ACE2‐(G4S)6‐Fc docked on S protein with three RBD domains in the “up” position. While ACE2‐Fc is expected to only engage one RBD, ACE2‐(G4S)6‐Fc is capable of engaging two RBDs (depicted in red). (c) Native‐PAGE of ACE2‐Fc (lane 2) and ACE2‐(G4S)6‐Fc (lane 3). (d) Yield of ACE2‐Fc and ACE2‐(G4S)6‐Fc after protein A affinity chromatography. Proteins were purified from 500 mL cultures of Expi293T cells. (e) Size exclusion chromatography of ACE2‐(G4S)6‐Fc and ACE2‐Fc. Figure S2. ACE2‐(G4S)6‐Fc effectively traps SARS‐CoV‐1 VLP in human AM. (A) Fraction of fast‐moving SARS‐CoV‐1 and (B) ensemble‐averaged effective diffusivities of SARS‐CoV‐1 in AM treated with various mAbs or ACE2 decoys. Figure S3. Biophysical characterization of nebulized ACE2‐(G4S)6‐Fc. (A) Native‐PAGE of nebulized ACE2‐(G4S)6‐Fc. Samples were collected from the upper chamber (lanes 2, 5, 8), lower chamber (lanes 3, 6, 9), and left‐over liquid (“dead volume”) after nebulization (lane 4, 7, 10) of the nebulization device. Data is shown for three repeats. (B) Size exclusion chromatography of ACE2‐(G4S)6‐Fc before nebulization and samples collected from the upper chamber, lower chamber, or left‐over liquid of the nebulization apparatus. Data representative of three repeats is shown. Figure S4. Differential scanning fluorimetry of ACE2‐(G4S)6‐Fc. Data for three independent repeats is shown in the figure. [file BTM2-9-e10650-s001.docx]

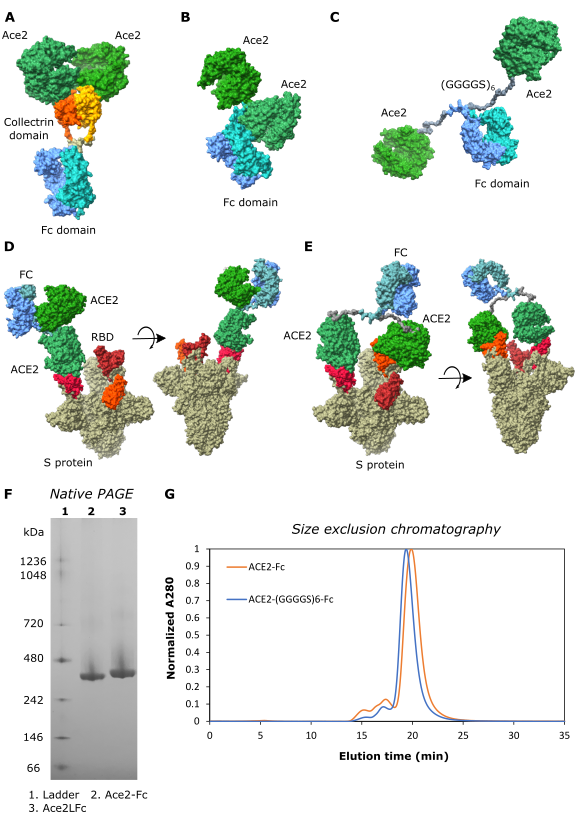


**ACE2-(G_4_S)_6_-Fc**

**ACE2-Fc**

**B B C**

**A B C**

**
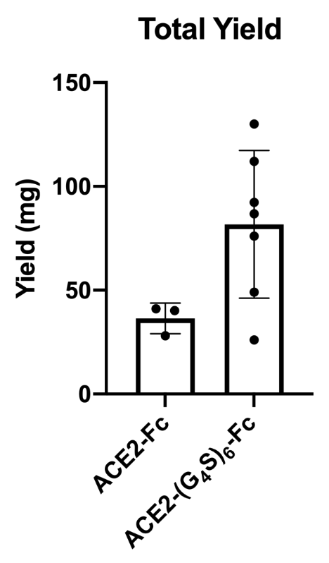
**

**E B C**

**D B C**

**C B C**


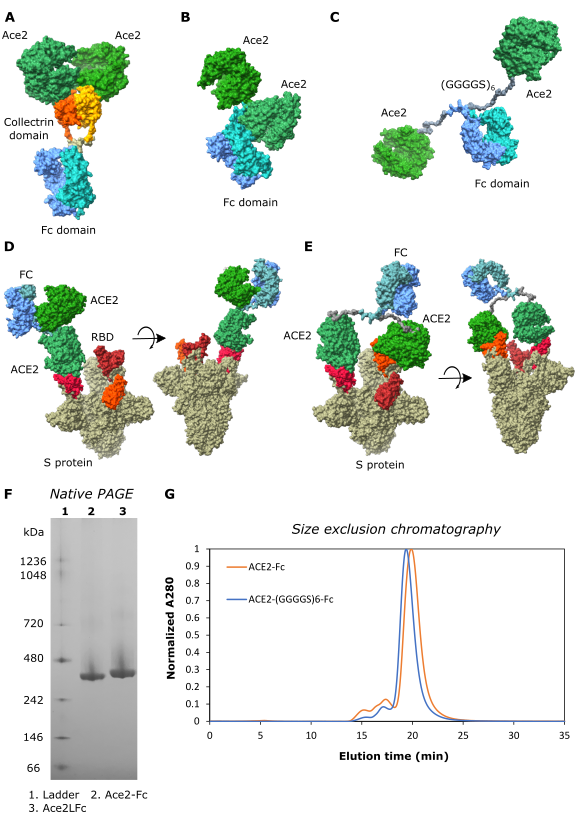

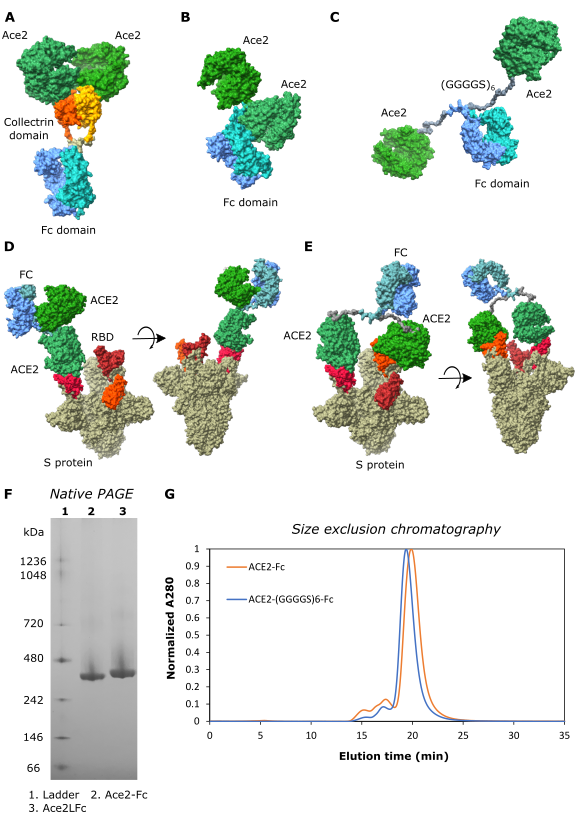


**1 Ladder**

**2 ACE2-Fc**

**3 ACE2-(G_4_S)_6_-Fc**

**Supp Figure 1. (A)** Computational prediction of **(A)** ACE2-Fc and **(B)** ACE2-(G_4_S)_6_-Fc docked on S protein with three RBD domains in the “up” position. While ACE2-Fc is expected to only engage one RBD, ACE2-(G_4_S)_6_-Fc is capable of engaging two RBDs (depicted in red). **(C)** Native-PAGE of ACE2-Fc (lane 2) and ACE2-(G_4_S)_6_-Fc (lane 3). **(D)** Yield of ACE2-Fc and ACE2-(G4S)6-Fc after protein A affinity chromatography. Proteins were purified from 500 mL cultures of Expi293T cells. **(E)** Size exclusion chromatography of ACE2-(G_4_S)_6_-Fc and ACE2-Fc.

**Supplementary Figure 2.** ACE2-(G_4_S)_6_-Fc effectively traps SARS-CoV-1 VLP in human AM. **(A)** Fraction of fast-moving SARS-CoV-1 and **(B)** ensemble-averaged effective diffusivities of SARS-CoV-1 in AM treated with various mAbs or ACE2-decoys.

**
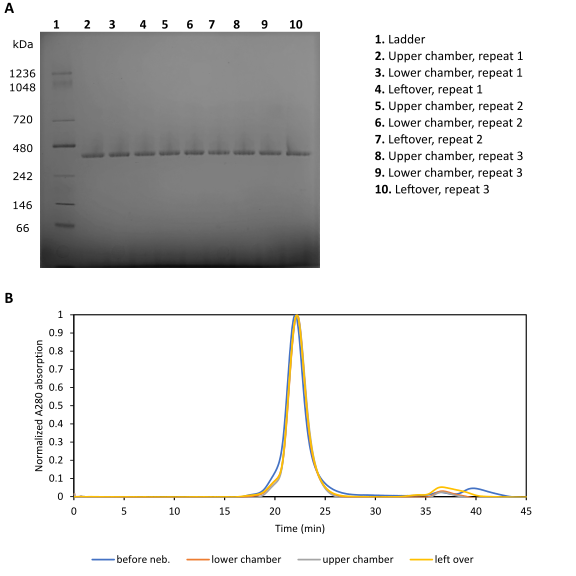
**

**Supplementary Figure 3. Biophysical characterization of nebulized ACE2-(G_4_S)_6_-Fc. (A)** Native-PAGE of nebulized ACE2-(G_4_S)_6_-Fc. Samples were collected from the upper chamber (lanes 2,5,8), lower chamber (lanes 3, 6, 9), and left-over liquid (“dead volume”) after nebulization (lane 4, 7, 10) of the nebulization device. Data is shown for 3 repeats. **(B)** Size exclusion chromatography of ACE2-(G_4_S)_6_-Fc before nebulization and samples collected from the upper chamber, lower chamber, or left-over liquid of the nebulization apparatus. Data representative of 3 repeats is shown.


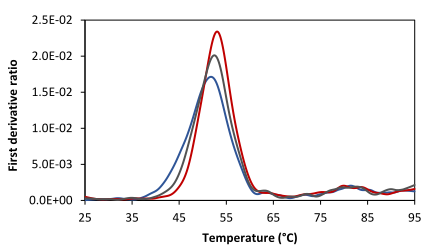


**Supplementary Figure 4. Differential Scanning fluorimetry of ACE2-(G_4_S)_6_-Fc.** Data for three independent repeats is shown in the figure.
